# A new approach for two-terminal electronic memory devices - Storing information on silicon nanowires

**DOI:** 10.1038/srep27506

**Published:** 2016-06-09

**Authors:** Konstantina Saranti, Sultan Alotaibi, Shashi Paul

**Affiliations:** 1 Emerging Technologies Research Centre, De Montfort University, The Gateway, Leicester, LE1 9BH, United Kingdom

## Abstract

The work described in this paper focuses on the utilisation of silicon nanowires as the information storage element in *flash-type* memory devices. Silicon nanostructures have attracted attention due to interesting electrical and optical properties, and their potential integration into electronic devices. A detailed investigation of the suitability of silicon nanowires as the charge storage medium in two-terminal non-volatile memory devices are presented in this report. The deposition of the silicon nanostructures was carried out at low temperatures (less than 400 °C) using a previously developed a novel method within our research group. Two-terminal non-volatile (2TNV) memory devices and metal-insulator-semiconductor (MIS) structures containing the silicon nanowires were fabricated and an in-depth study of their characteristics was carried out using current-voltage and capacitance techniques.

Over the last a few decades a significant progress in the field of electronic memory is noticed, mainly due to a rapid increase in the use of handheld devices such as smartphones, e-readers and tablets. The main objective, specifically in non-volatile class of memory devices, is to reduce the cell memory size while increasing the storage density. However, miniaturisation of exisiting complementary metal oxide semiconductor (CMOS) based memory technology will soon reach its limit due to serious scaling challenges^1^,^2^. Currently, alternative manufacturing and processing equipment as well as materials and designs for the storage devices are being proposed. Another key aspect that should be taking into account is the thermal budget and in particular the reduction of the fabrication temperatures that will lead to a reduction in carbon footprints and cost.

Following this demand, different types of memories such as polymer, phase change and resistive have been reported^3^,^4^. Organic memories appear to be of great interest because of their simple structure and three-dimensional (3D) capability^5^. It has been also described that organic molecules as well as gold nanoparticles in a polymer matrix can exhibit non-volatile memory effect. The memory behaviour, in aforementioned devices, is attributed to a charge transfer complex formed between the polymer material and the nanoparticles^3^,^6^. However, because of the organic nature, there are few drawbacks such as contamination, parasitic conduction paths, temperature stability and incompatibility with silicon-based technology. Silicon (Si) is preferred in semiconductor industry as it is compatible with the current fabrication technologies. More than ever, Si materials at nanoscale engrave a new path to semiconductor industry, mainly due to their extraordinary physical and chemical properties in comparison to their bulk properties. Among them, silicon nanowires (SiNWs) are the most promising due to the fact that they are one-dimensional (1D) semiconductors and the quantum mechanical properties become significantly important for such structures^7^. In addition, SiNWs have already been used as building blocks as well as interconnectors for a wide variety of electronic devices such as field effect transistors, solar cells and memory devices^8^,^9^,^10^. Since there is a need in the field of electronic memory devices, which combines low cost, high performance and long retention times; in this paper, a study of the SiNWs as the storing component of a two-terminal non-volatile (2TNV) memory devices is studied.

In non-volatile memories, the elapsed time between data storage and the first invalid readout of the data is the retention time^1^. Each non-volatile storage technology employs a particular storage mechanism and material properties related to that mechanism. Its implementation format will verify the retention features of the device. For flash memories, the storage mechanism is to represent data by quantities of charge held on a floating gate. Each technology can be expected to have some natural processes where the data retention capability changes with time. Non-volatile and flash has some intrinsic charge decay characteristics that define the ultimate retention potential of the approach. At the present time, a typical retention time specification is 10 years. Defects related to the materials, details of device geometry or aspects of circuit design could impact the retention time. Each of these three potential problematic areas may result in the addition or removal of charge to/from the floating gate, causing the threshold voltage to be shifted. Gate insulator defects are typical causes of retention degradation. Other mechanism(s) associated with ionic contamination or traps can also be the contributing factors. Reliability of memory devices, in particular program/erase endurance and data retention, is a key issue of flash technology.

The working principle of the 2TNV memory presented in this article is based on the model proposed by Paul, S. wherein the realisation of the non-volatile memory behaviour is attributed to the creation of an internal (or surplus) electric field when a voltage is applied across the device^11^. The value of this electric field will define the difference between the two distinctive conductivity states^11^. A preliminary demonstration of the use of silicon nanostructures for the storage element has been presented by our group^12^.

In this work, a 2TNV memory device, using SiNWs as an active layer, exhibiting electrical bistability is presented. The device structure has a charge storage medium (that is the “silicon nanowires”) which is sandwiched between two dielectric layers with metal top and bottom contacts as shown in Fig. 1. When a certain electric potential is applied across the device, the electric conductivity of the device in discussion is changed. The change in the electrical conductivity is further examined for the non-volatility, if any. Figure 1 also shows the schematic of expected current-voltage response of the 2TNV memory device based on the proposed hypothesis. A high write/erase voltage and a read voltage that is low enough to monitor the state, but not too high to cause any physical damage are applied. At a write voltage (point 1), the electrons have tunnelled through the insulator and get trapped into the SiNWs. At the read state (point 2), a decrease in conductivity has been monitored since the effective voltage across the device is less than that of the applied voltage (E_o_). After the erase process (point 3), a different red state (point 4) is observed, indicating that the direction of internal field (E_i_) has altered.

Data retention measurements will demonstrate the period in which the state of the memory is retained. While write-read-erase-read measurements for the high number of pulses will also show that the proposed memory device has endurance capability. Impurities and/or trapped charges may affect the performance and working mechanism of proposed device; an investigation has also been undertaken to comprehend such influence on working of device, if any.

## Materials and Methods

The device is made of SiNWs, which are sandwiched between dielectric layers on the glass substrate with aluminium (Al) bottom and top contacts. Corning 7059 glass substrates with 100 nm evaporated Al tracks for the bottom contacts have been used as starting substrate. Thermal evaporation was used for the Al tracks. The dielectric layers (silicon nitride,) as well as the SiNWs were deposited by Plasma Enhanced Chemical Vapour Deposition (PECVD) technique with a 13.56 MHz RF PECVD reactor.

The silicon nitride thin film layers were deposited using SiH_4_, N_2_ and NH_3_ gases at 300 °C, chamber pressure of 350 mTorr and RF power of 20 Watts. SiNWs for the 2TNV memories as well as the MIS devices were deposited using previously published method from our research group^13^; Ga catalyst layer was deposited before PECVD process; temperature of 400 °C, a pressure of 200 mTorr, RF power of 25 Watts and growth time of 30 minutes. SiH_4_ and H_2_ flow rates were set to 20 and 100 sccm respectively. Before introducing the SiH_4_ gas, H_2_ plasma was active for 5 minutes. Following the second dielectric layer, finally 100 nm Al top contacts were deposited to complete the two-terminal device cell. A reference sample was also fabricated with the same deposition conditions without the catalyst.

The metal-insulator-semiconductor (MIS) capacitor structures were fabricated on p-type Si substrate, which had an ohmic back contact. Spin coating was used for the deposition of the insulating layers. First polystyrene (PS) and then polyvinyl acetate (PVAc) were spin coated forming an insulator layer of 100 nm thickness. SiNWs with similar deposition conditions as earlier were used; temperature of 250 °C, a pressure of 200 mTorr, RF power of 25 Watts, growth time of 10 minutes and SiH_4_ flow rate 20 sccm. At the end, 50 nm Al were thermally evaporated for the top contacts. The MIS structures were used in order to understand the charging behaviour of SiNWs. Reference samples for the memory cell as well as the MIS devices were also prepared with the same deposition conditions, but with no catalyst material and hence no SiNWs. The 2TNV memories have an area of 0.01 cm^2^ and the MIS structures an area of 0.017 cm^2^. Both have approximately 1000 nm vertical dimensions.

Scanning electron microscopy (SEM) analysis was conducted to investigate the spatial of distribution of the SiNWs on the substrate. Current-Voltage (I–V), data retention time (Current-Time), write-read-erase-read (W-R-E-R) measurements were conducted using HP 4140B pico-ammeter and Capacitance-Voltage (C–V) measurements of MIS capacitors were conducted using an LCR bridge HP 4192A with a frequency of 1 MHz. All the electrical measurements were conducted at room temperature and in an electromagnetic shielded dark box.

## Results and Discussion

### Analysis of the 2TNV memory with SiNWs as the floating gate

**Figure 1:**
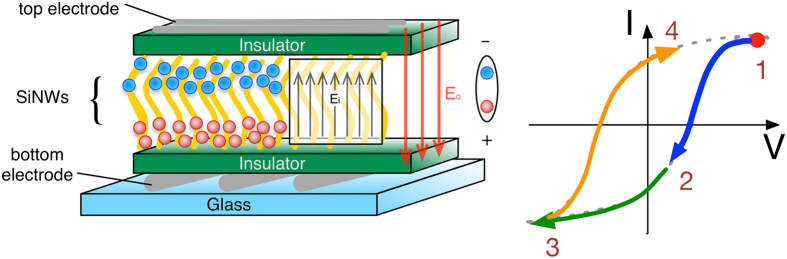
Schematic diagram presenting the two-terminal memory (2TNV) device in the READ process, explaining the formation of the negative internal electric field E_i_ and the current-voltage plot based on the working principle of the 2TNV memory.

**Figure 2:**
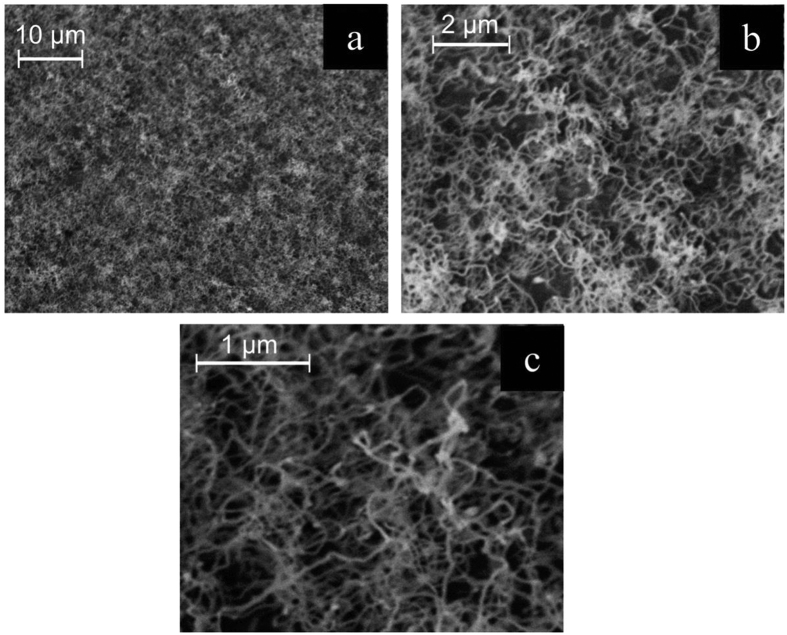
SEM images of SiNWs growth at 400 °C with a magnification of (**a**) 1.00 k, (**b**) 5.00 k and (**c**) 10.00 k showing spaghetti-like growth of silicon nanowires.

The proposed memory device consists of SiNWs sandwiched between dielectric layers and a cross-point array of top and bottom contacts on glass substrate as shown in Fig. 1. The area where the top and the bottom contacts cross is a memory cell. The reference sample has a similar configuration with a layer of amorphous Si (a-Si) instead of the SiNWs. If we do not use any metal catalysis, only amorphous Si deposition occurs under the same growth conditions. The nature of this layer was verified by Raman spectroscopy (data not shown) as well as SEM analysis. The main purpose of the reference (or control) sample is to provide information whether the electrical bistability is arising from insulating layer and SiNWs of the device solely or together. The morphology and the uniformity of the SiNWs were studied with SEM. Figure 2 shows SEM images of the SiNWs at different magnifications. It is evident from the SEM micrographs that the spatial uniformity of SiNWs across the substrate is acceptable to realise memory devices. I–V characteristics of both the reference sample and the active sample (termed as 2TNV memory device) were obtained. The hysteresis of the I–V characteristics indicates the capability of the device to store charges. If we are able to observe a reliable electrical bistability in our fabricated devices, we can use them to represent the binary system (0–1 as high-low states).

**Figure 3:**
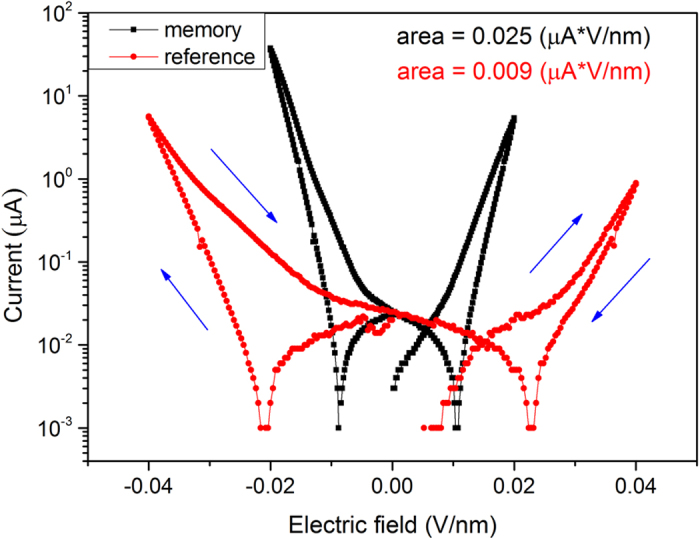
Log (I)–V characteristics of the 2TNV memory using SiNWs for the storage medium (black line) and the reference sample with no SiNWs (red line). It is obvious that there is a difference between the two samples.

As stated earlier, the hysteresis observed in electrical measurements (C–V and I–V) is a crucial indication of charges are being stored. A measured value of 0.025 μΑ/nm of the area enclosed (hysteresis) was measured for the novel 2TNV memory with SiNWs while a relatively small value (0.009 μAV/nm) for the reference sample. The I–V curve is slightly asymmetrical due to interfaces between the different layers such as the silicon nitride and the Al contacts. This hysteresis is worth mentioning especially in comparison to the reference sample with the a-Si layer (Fig. 3, red line). Since the reference sample was fabricated in tandem with device containing silicon nanowires; the SiNWs are credited for the higher charge trapped. On the basis of this observation, we believe that there is greater charge storage when SiNWs are present. Moreover, the insignificant hysteresis of the reference sample (0.009 μAV/nm) points out that there is a very small amount of impurities and defect states.

By applying negative or positive voltage the electrical conductivity of devices containing SiNWs is appreciably changed. When voltage is applied across the device, a significant proportion of electrons will flow through the insulator layer and then these electrons will be stored in SiNWs. This causes the creation of a negative internal electric field (E_i_). For the read process a lower positive voltage is applied. In that case, the voltage across the device is less than that of the applied voltage resulting in the decrease of the conductivity. The high voltage of the opposite polarity will reverse the process (erase operation) and create a positive internal electric field. Because the insulator layer is quite thick, a high voltage has to be applied. The value of the electric field is a crucial parameter, which will determine the difference between the two distinct conductive states.

**Figure 4:**
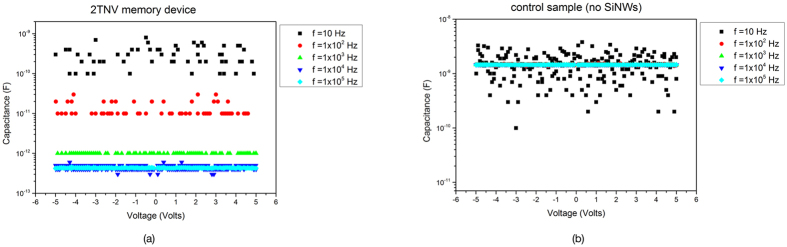
Capacitance-voltage data at different frequencies for the 2TNV memory device and the control sample. It is evident that the control sample shows no noticeable difference while for the 2TNV memory the capacitance is decreasing with increasing the frequency.

**Figure 5:**
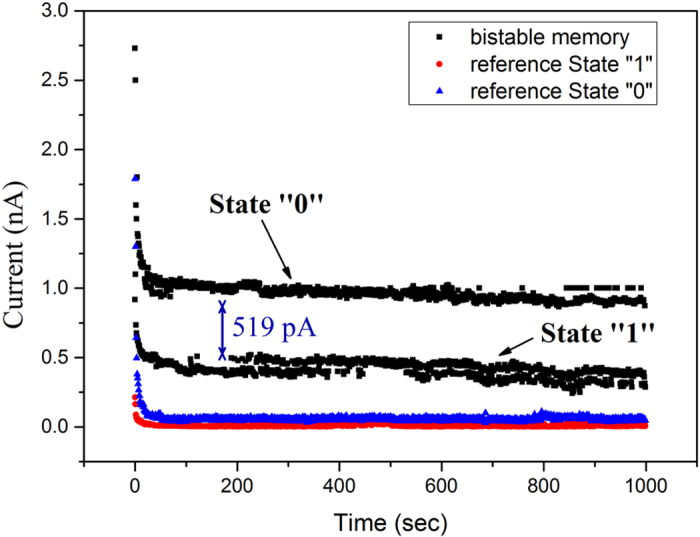
Data retention time for the bistable memory based on SiNWs (black) and the reference sample (red-blue). Write at +25 V, read at +7 V for 10^3^ pulses per second. Two distinguished electrical conductivity states are formed; a high conductivity state (State “0”) and a low conductivity state (State “1”). The reference sample presents also two stable states with a minute magnitude difference in the current.

In order to further consolidate our argument that the SiNWs are exhibited storing capability, capacitance-voltage behaviour as a function of frequency of 2TNV devices and a control sample were investigated. Capacitance-Voltage sweeps with different frequencies at constant temperature were performed and the observed behaviour is presented in Fig. 4. The deep level defects (or traps), within an energy band gap of semiconductor materials, can only respond to low values of frequency of C–V measurements. The high-frequency capacitance does not include any contribution from deeper defects. The results presented in Fig. 5 come in agreement with our hypothesis that the SiNWs are credited for the memory effect. The capacitance as a function of frequency for the MIM capacitor with no SiNWs (control sample) does not show any significant change in the capacitance as a function of frequency. The capacitance of 2TNV was found to decrease with increasing the frequency. Hence, the electrons/charges are predominately captured in SiNWs but not in silicon nitride dielectric layers.

Furthermore, the electrical bistability is examined; two different conductivity states-high and low-are detected. Since it is important to know if the difference in current (or in other words maintaining two discernable conductivities states) can be retained. The retention time measurements were carried out highlights that data can be retained for a certain period of time. The observation of the distinct difference in the two states is evidence for the memory behaviour. The difference in value of current is not so vital as long as it does not significantly vary with time and the two states is distinguishable and stable over time. The data retention behaviour of the memory device and of the reference sample was tested. It was written at +25 V and read at +7 V for 10^3^ pulses.

Figure 5 shows the bistable memory behaviour of our memory devices. The initial sharp decrease of the current is attributed to capacitance phenomena of the system. After the first 30 pulses, a stable behaviour of the device is observed. These two stable states “0” and “1” are similar to binary bits for information. It is proven that for 10^3^ read pulses these states are stable with a current difference 519 pA. Although the reference sample follows the same behaviour (Fig. 5), the difference between states “0” and “1” for the reference sample gives a value of 50 pA (as ~520 pA in devices containing SiNWs). This small difference may arise from defects in silicon nitride and/or amorphous Si. The two-terminal memory device based on SiNWs has 10 times higher difference in two states than the reference sample, leading to the conclusion that the SiNWs can be used as a non-volatile memory device.

**Figure 6:**
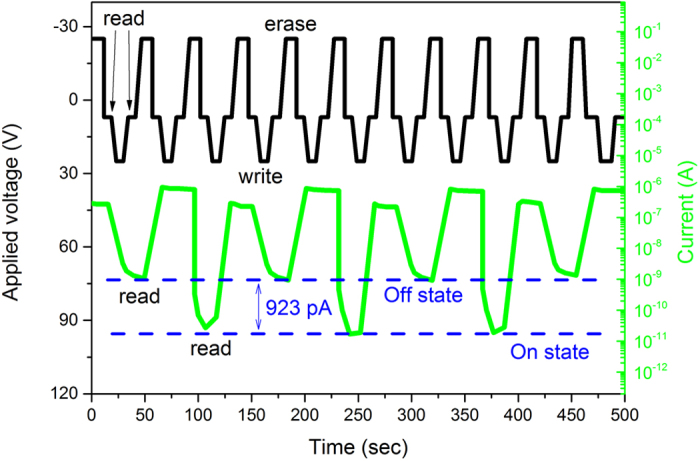
Write-read-erase-read characteristics of the 2TNV memory devices. The current (green) versus voltage (black) for a period of pulses was monitored showing a current difference between the ON and OFF states.

The write-read-erase-read the characteristics of the 2TNV memory device were studied. The results are shown in Fig. 6. The current response (green) to the applied voltage (black) is monitored for a number of pulses. First +25 V were applied to the device (write operation) followed by +7 V read voltage and the device switched to a low conductivity state, ON. When −25 V were applied, the device switched to a high conductivity state, OFF state, again reading at +7 V. It is noticeable a current difference of 923 pA at the read process between the ON and OFF state. This demonstrates that our memory is rewritable. Similar behaviour was observed for most of our two-terminal memory devices for a period of 10^3^ pulses.

The noise level maximum value is 3 pA, measured for 0 Volts applied to the device. The switching current range at the read voltage is 500~1000 pA. In particular, there is a 519 pA difference for the retention time data (see Fig. 5) and a current difference of 923 pA between 1 and 0 state for the write-read-erase-read characteristics (see Fig. 6). The presented data are obtained for 2TNV memories with an area of 1 mm^2^. These results provide a proof of concept that the proposed hypothesis (storing charge on silicon nanowires) and its methodology are applicable. Since the switching current will be scaled with decreasing the device size, further improvements need to be made such as alignment and reduction of the length of the SiNWs that could improve the memory characteristics (i.e higher switching current) and provide a better quality device. The reduction in the thickness of insulating layer will also increase the current through the device. Therefore, a combination of length of silicon nanowires and their uniformity over a substrate with the thickness of insulating layers will pave a path to scale down the device features while the values of current (for both ON and OFF states) are still higher than thermal noise or lower limit of the current sensing element. We can confidently say that the basic principle presented here is valid and the novelty of this work is related to a fundamental advances in control and understanding of silicon nanowires in electronic memory devices. To the best of our knowledge, it is for the first time that SiNWs are established as a charge-storing component for two terminal memory devices.

### Analysis of MIS structures

**Figure 7:**
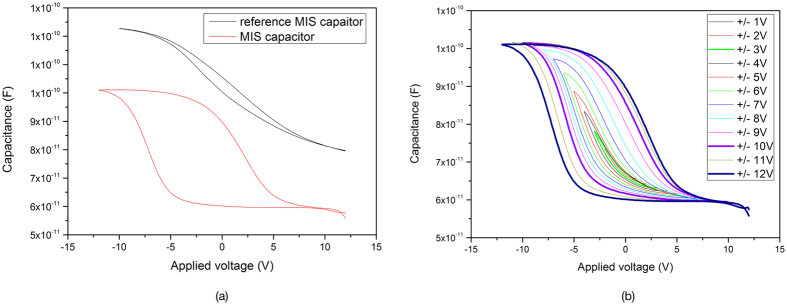
(**a**) Capacitance-Voltage characteristics of the reference (p-Si/PS&PVAc/a:Si/Al) device (black) and MIS capacitor (p-Si/PS&PVAc/SiNWs/Al) devices (red). It is obvious that the MIS cell presents a significant large hysteresis in comparison to the reference sample. (**b**) Capacitance-Voltage measurements of MIS capacitor at 1 MHz with various scan ranges of bias (from ±1 V to ±12 V). The accumulation, depletion and inversion regions of the capacitor are observed as well as the increase of the width of the hysteresis-area by increasing the voltage.

A further study of the charging properties of the SiNWs was conducted by fabricating MIS capacitor structures (Al/PS&PVAc/SiNWs/p-Si with native oxide/Al). Equivalent to previous devices a reference sample with the same configuration, but no SiNWS was prepared. The leakage current of the dielectric film PS-PVAc reached a maximum value of 50 pA indicating a reliable electrical insulator. High frequency (1 MHz) multiple C–V sweeps between inversion and accumulation region were performed for the MIS capacitor and a reference sample in order to examine the charge trapping characteristics. The properties of these curves can be explained due to the capacity of storage and trap/detrap processes of charge carriers in SiNWs. C–V characteristics of the reference and MIS capacitor devices are shown in Fig. 7. The reference sample presented a negligible small hysteresis, indication of high quality layer, while the MIS capacitor with SiNWs (Fig. 7(a), red curve) has a larger hysteresis.

Figure 7 shows the C–V curves of the same MIS capacitor where the bias voltage was increased and swept from ±1 V to ±12 V. This meant that bias swept from the accumulation region into the inversion region for a p-type MIS capacitor. The broadening of the hysteresis loop and hence the area that encloses is observed and the correlation of it to the amount of charged stored is studied thoroughly.

The density of trapped charges per area, n, can be calculated using the equation (i); where Cacc is the capacitance at accumulation, ΔV_FB_ is the difference in flatband voltage shift for the forward and reverse scan (ii), A is the gate area and q the magnitude of the electron charge.


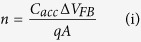


Where,


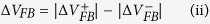


**Figure 8:**
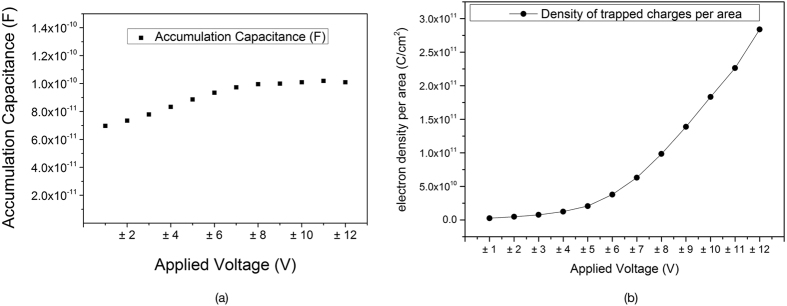
(**a**) Capacitance at accumulation (Cacc) for various sweep voltage ranges. A stable behaviour is observed, especially for higher voltages in which the accumulation region is t strongly taking place. (**b**) The density of trapped charges per unit area for the forward and reverse scan. All data are extracted and calculated based on the Capacitance-Voltage behaviour of the MIS devices.

At the first scan between −1 Volt and +1 Volt (±1 V scans), an insignificant hysteresis was found, indicating negligible charge trapping in SiNWS, which is in agreement with the calculated density of trapped charges per area (2.57E + 9). The accumulation capacitance showed substantial stable behaviour as it can be seen in Fig. 8(a). Hence the change in flatband voltage shift will be the dominant factor for the change of the density of trapped charges. Since more electrons were injected at ±12 V scan compared to ±6 V, a larger V_FB_ was observed for the ±12 V scan. Consequently, the amount of trapped charges per area and the density is much greater for higher voltages and is confirmed by the data shown in Fig. 8(b).

**Figure 9:**
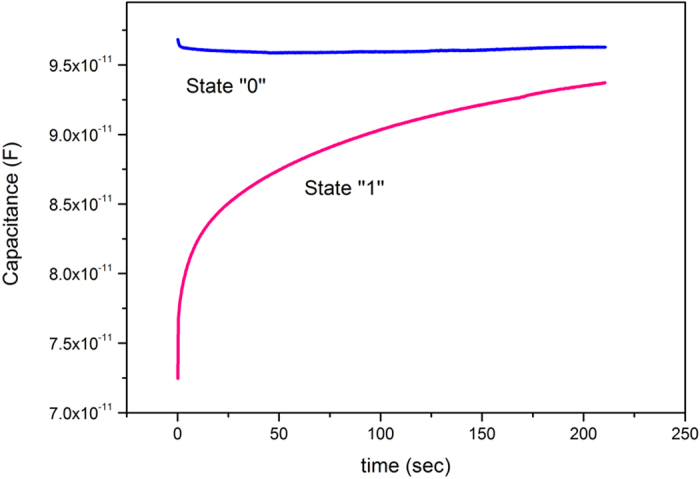
Retention time of MIS capacitor with SiNWs showing 10^3^ read pulses over a period of around 40 minutes. The two states remained stable after an initial gradual decay in one state.

The retention characteristics were also examined so that the reliability of the structure could be tested. Write and erase voltages of ±10 V and a read voltage of −5 V for 10^3^ pulses were applied. Over this time span, the states 0 remained stable and while the state 1 is a gradual decayed (shown in Fig. 9).

## Conclusions

The research conducted in this paper was primarily concerned with the investigation of the SiNWs a charge storing component for future flash memory-type devices. The charging of SiNWs (grown at low temperature) can be appealing to the community working in the area of Nanoscience. Our approach aims to reduce the cost and the temperature budget as well as suggest a way to scale down the dimensions of the memory cell. A full analysis of 2TNV memory devices and MIS capacitors containing the SiNWs was conducted. The I–V behaviour showed a significant hysteresis in comparison to the reference sample. The capacitance measurements, as a function of frequency, indicate that the defect states are present in SiNWs. The electrical measurements, of devices fabricated using SiNWs presented in this paper, are clear indications of memory behavior. Write-read-erase-read pulses were also generated showing the ability to rewrite for many pulses. MIS capacitors were studied in order to further analyse charging and discharging phenomena and a correlation between the hysteresis and the density of stored charges was also established. Using the MIS structure the carrier injection mechanism of the capacitor could be verified by sweeping it through different voltage ranges. Furthermore, the capacitor with the SiNWs showed a large hysteresis, which can be credited to charge storage in the SiNWs. In a nutshell, it was proven that these devices were able to hold charges and displayed memory behaviour.

## Additional Information

**How to cite this article**: Saranti, K. *et al.* A new approach for two-terminal electronic memory devices - Storing information on silicon nanowires. *Sci. Rep.* **6**, 27506; doi: 10.1038/srep27506 (2016).
